# Enhanced Corrosion Protection Performance by Organic-Inorganic Materials Containing Thiocarbonyl Compounds

**DOI:** 10.1038/s41598-018-29299-5

**Published:** 2018-07-19

**Authors:** Wail Al Zoubi, Young Gun Ko

**Affiliations:** Materials Electrochemistry Group, School of Materials Science and Engineering, Yeungnam University, Gyeongsan, 38541 Republic of Korea

## Abstract

In the present study, the synergistic effect on the corrosion protection properties of Mg alloys subjected to plasma electrolytic oxidation and chemically treated with thiourea as an inhibitor is investigated by surface microstructure analysis, evaluation of the electrochemical performance, and chemical quantum calculations. Physical adsorption of thiourea on the inorganic material surface might be due to physical interaction between thiourea with a low ionization potential serving as an electron donor and the inorganic components with high electron affinities acting as acceptors. The results from potentiodynamic polarization and electrochemical impedance spectroscopy for organic-inorganic coating reveal a clear decrease in the corrosion rate owing to the introduced thiourea.

## Introduction

As the lightest materials, magnesium alloys have been used in a broad range of applications with promising futures in corrosion science, automobiles, biomedicine, computer parts, and electronic industry, owing to their low density, high specific strength, castability, machinability, and high thermal conductivity^1^. However, poor corrosion and wear resistance and high chemical reactivity of magnesium constitutes have limited the widespread use of Mg alloys in many practical applications^2–4^.

Plasma electrolytic oxidation (PEO) is an innovative technique to improve the anti-corrosion ability of Mg and its alloys. It is considered one of the most effective ways to improve the properties of light-weight metallic materials and to impart new functionalities as well as improve the adhesion of the subsequent coating^5^. Moreover, coating layers obtained by PEO could be oxidized by a corrosive medium because many microdefects existing in the oxide surface might facilitate quicker penetration of the chloride ions into the base magnesium^6,7^. Other methods to improve the protective performance of coating layers formed by the PEO process are pre-deposition, polymer coatings, sol-gel approach, electroless metal deposition, and dip-coating (DC)^8–10^. Among these approaches, the surface modification of the coatings obtained by PEO followed by DC with another material is one of the most promising for blocking porous materials and forming a chemical-conversion coating for electrochemical applications^11–15^. However, the mechanism of how the organic or inorganic component influences the corrosion process was investigated. Owing to their morphological structure, coating layers obtained by PEO could be used as a base coating for the fabrication of composite coatings, that could serve various functional purposes and also contain a corrosion inhibitor in its chemical composition^16–23^. For instance, Ping *et al*.^24^ reported that the corrosion protection performance could be enhanced by blocking the microdefects on the surface of the coating obtained by PEO using poly lactic acid (PLA). Malayoglu *et al*.^25^ reported that blocked 10-µm thick PEO coatings show better corrosion performance than 25-µm thick unblocked PEO coatings. Meanwhile, Zhang *et al*.^26,27^ prepared PEO coatings on AZ91 HP in an electrolyte containing phytic acid (C_6_H_18_O_24_P_6_) and reported that the addition of phytic acid into base solution aids the formation of the coating and that the coatings exhibit the best uniformity and corrosion protection. However, organic components containing several heteroatoms (i.e., nitrogen, oxygen, and sulfur) could hinder the corrosion of metals by interacting with the coating surface via chemisorption through electron cloud of the electron-donating groups of the molecules^25^. The chemical stability of the adsorbed organic layer on the coating surface depends on the surface charge of the light-weight metallic material, the chemical structures of the organic compounds and the type of the corrosive medium^28,29^. Among various organic compounds, thiourea (CN_2_H_4_S) is used as a corrosion inhibitor for metals and their alloys^30,31^. Moreover, the lone pairs of electrons in the amino and thiocarbonyl groups could facilitate the adsorption of thiourea on the inorganic coating surface and thereby lead to provide a higher inhibition performance.

Despite numerous studies, there are no reports on the synergism of an organic component, such as thiourea, and the coating obtained by PEO on AZ31 Mg alloy significantly enhancing the anti-corrosion properties of the metal alloy surface. Moreover, recently, there is significant interest in studying PEO technique to prevent or stop the corrosive solution from reaching the light metals and their alloys. In this study, PEO and chemical treatment by thiourea were combined to prevent the corrosion of Mg. Apart from, the efficiency of thiourea as an organic inhibitor on the coating surface was investigated by potentiodynamic polarization (PDP) and electrochemical impedance spectroscopy (EIS). We also aimed to study the effect of the structure of the inhibitor on the inhibition efficiency and study the mechanism of inhibitor adsorption the coating surface and hence correlate the experimental results with that of the quantum chemical calculations on the prepared inorganic surfaces.

## Methods

Samples of the magnesium AZ31 alloy (mass fraction: Al; 3% Zn; 1%; balance) were used as substrates in this study. Before the PEO treatment, every surface of the AZ31 Mg samples was successively ground and polished in water with SiC sandpapers of increasingly finer grains, i.e., 400, 800, 1000, 1200, and finally 2400 grit, rinsed with deionized water and then cleaned ultrasonically with ethanol. PEO process was carried out in an electrolytic cell containing 2 g L^−1^ hexamethylenetetramine ((CH_2_)_6_N_4_); HMT), 4 g L^−1^ KOH, 8 g L^−1^ NaAlO_2_, and 4 g L^−1^ glycerol (C_3_H_8_O_3_) in distilled water. The electrolyte temperature was maintained at 283 K to stabilize the electrochemical reactions throughout the PEO process. The PEO treatments were carried out for 6 min under an AC (alternative current) of 100 mA cm^−2^ and electronic power frequency of 50 Hz. The pH and electrical electrolyte conductivity of the aqueous electrolyte were ~14 and ~20 mScm^−1^, respectively.

After the PEO process, the treated samples were immersed in a 0.2 M solution of the organic inhibitor, thiourea (H_2_NCSNH_2_), in ethanol for 20 h. The surface morphology was observed by scanning electron microscopy (SEM-HITACHI, S-4800). The program Image Analyzer 1.33 was used to measure and calculate the pore size and the proportion of pores on the surface of the coatings. The constituent phase structures were analyzed by X-ray diffraction (XRD, RIGAKU, D-MAX 2500) with with Cu *Kα* radiation. The XRD patterns were collected with a step size of 0.05° over the scan range of 20° to 90°. Fourier transform infrared spectra (FT-IR; Perkin Elmer Spectrum 100) collected in the range of 400–4000 cm^−1^ were used to determine the polar functional groups on the remedied surface of the obtained film. The electrochemical measurements were performed at a scan rate of 1 mV s^−1^ in a 3.5 wt% aqueous sodium chloride (NaCl) solution at 25°C using an Ag/AgCl electrode in potassium chloride solution and a platinum foil as the reference and counter electrodes, respectively. Further, the corrosion behavior of the treated samples was evaluated by electrochemical tests (Gamry Instruments). The polarization curves were fitted and analyzed carefully from −0.3 to 0.4 V with respect to the open circuit potential (OCP) at a scan rate of 1 mVs^−1^. Electrochemical impedance spectroscopy (EIS) was conducted for the frequency range of 10^6^ to 0.1 Hz at an interval of 10 points/decade with a 10 mV rems. Further, quantum chemical analysis was performed using the Gaussian 03 W software, as described in ref.^32^.

## Results

### Microstructure characterization

**Figure 1:**
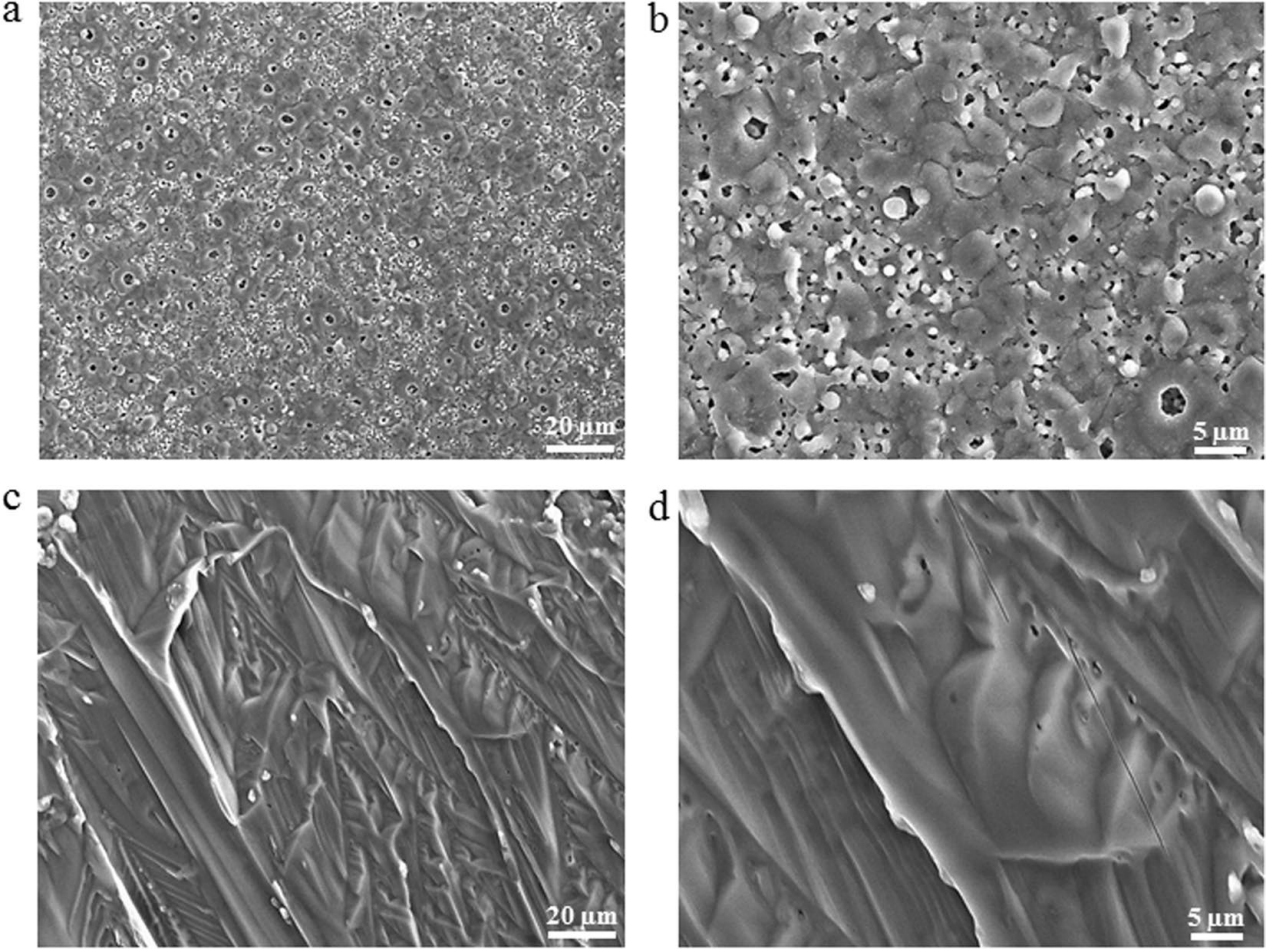
Scanning electron micrographs showing structural defects such as micropores and cracks in the samples treated by PEO coatings (**a**,**b**) and surface morphologies of the coating obtained followed by PEO followed by dip-coating with thiourea for 20 h at ambient temperature (**c**,**d**); the images figures are shown at various magnifications.

The surface morphology of the inorganic coating obtained by PEO followed by DC with thiourea is shown in Fig. 1. Figure 1a,b shows that microdefects are dispersed on the inorganic surface. Further, the PEO coating obtained in the presence of HMT consists of microdefects, but the organic layer (Fig. 1c,d) consisting of thiourea is uniform and smooth and it could overlay most of the micropores and microcracks on the surface of the PEO coating. The improved morphology after treatment with the organic component could be attributed to the polarity of the thiourea as well as the high electron donating abilities of nitrogen and sulfur atoms^13,24,31^. These findings indicate that the morphological structure of the PEO coating could be modified by thiourea coating.

### Chemical analysis of the coating layers

**Figure 2:**
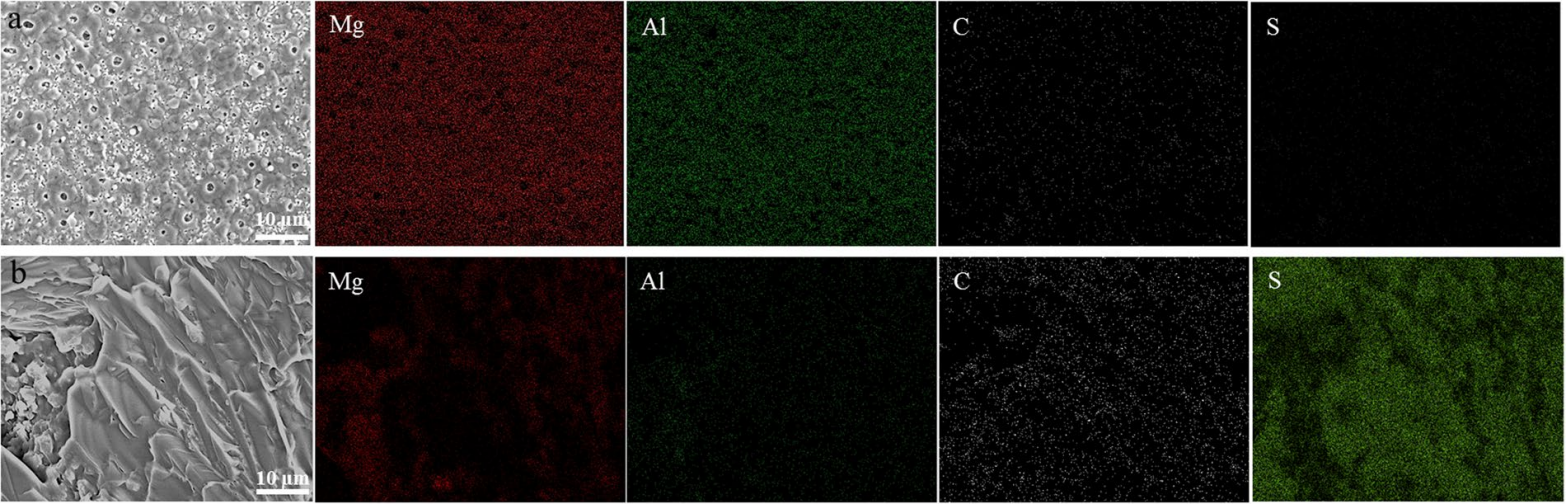
Surface morphology of AZ31 magnesium coated by (**a**) plasma electrolytic oxidation (PEO) at a current density of 100 mA/cm^2^ for 5 min and (**b**) PEO process followed by dip-coating in thiourea solution for 20 h at ambient temperature, and the elemental color maps of Mg, Al, C, and O.

**Figure 3:**
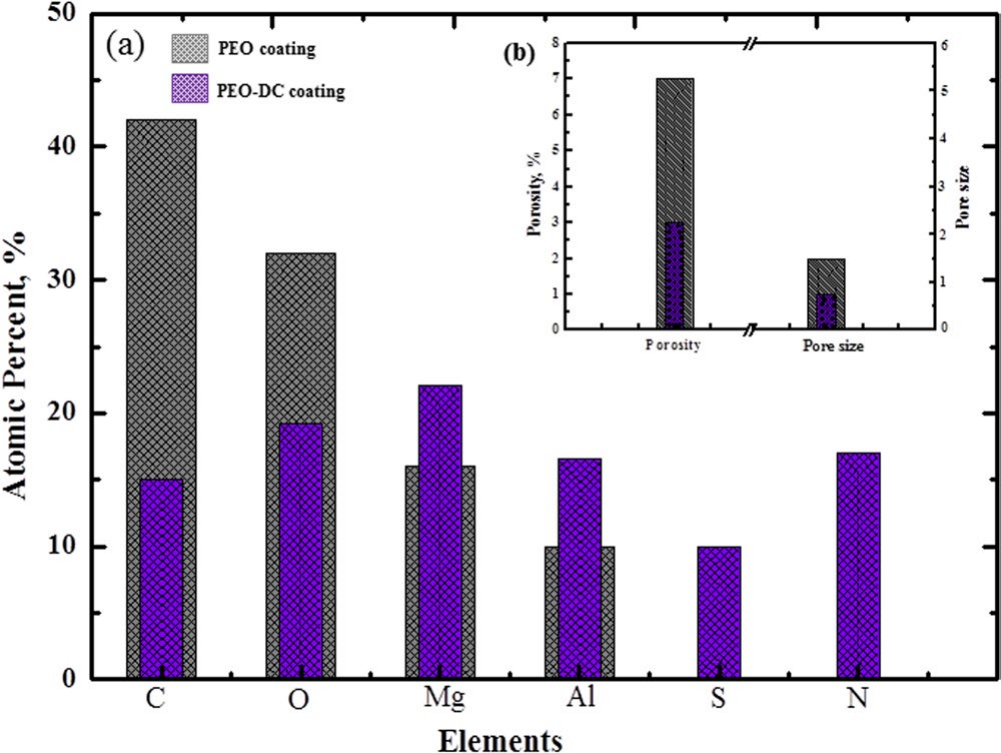
(**a**) Relative contents of chemical elements on the surface of the plasma electrolytic oxidation (PEO), and PEO coating followed by dip-coating (DC) with thiourea; and (**b**) characteristics of the surface porosity and pore size of PEO coating and PEO-DC coating.

The EDS analysis shown in Fig. 2(a) indicates that the C, N, and S contents of the treated samples increase in comparison with the PEO coating owing to the presence of thiourea in the organic layer on the inorganic surface. Moreover, the treated samples show decreased content of Al and Mg elements, revealing the existence of a thin thiourea-rich layer on the surface of the inorganic layer. In this regard, EDS elemental mapping of the coating layers (Fig. 2) confirms the presence of carbon and sulfur in the organic-inorganic materials. The presence of carbon and sulfur in the organic layer is due to the thiourea adsorbed on the inorganic layer owing to electrostatic attraction between the electron-donating thiourea molecules and the porous surface of the inorganic layer. The increase in the S content and decrease in the Mg and Al contents are attributed to the thiourea layer formed on the inorganic coating surface (Fig. 3a).

**Figure 4:**
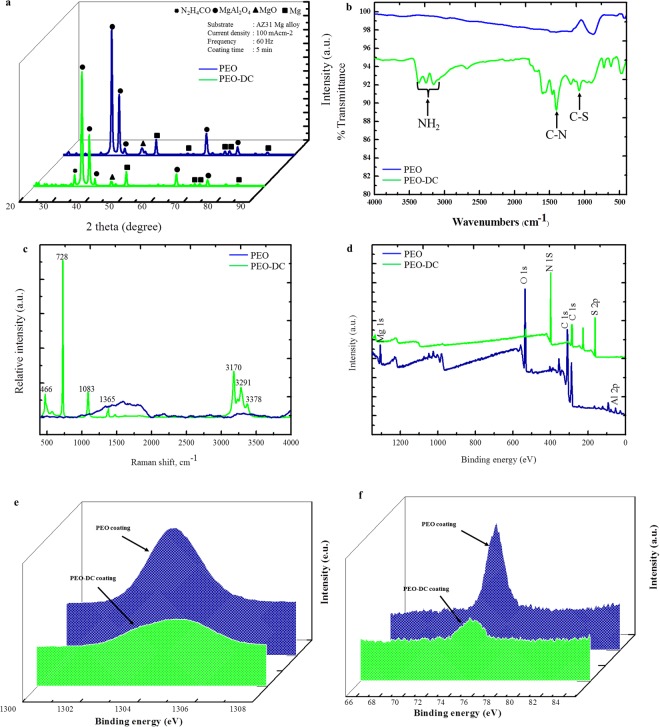
(**a**) XRD patterns of the samples coated by plasma electrolytic oxidation (PEO), and samples coated by PEO process followed by DC with thiourea. Scan range, are from 20 to 90°; Cu Kα radiation source. (**b**) FT-IR spectra of the coated materials with and without thiourea. (**c**) Raman spectra of the (plasma electrolytic oxidation) PEO coatings and PEO coatings followed by dip-coating (DC) with thiourea. (**d**) Full XPS spectra of the PEO coatings and PEO coatings followed by DC with thiourea. Normalized, high-resolution XPS scan of (**e**) Mg and (**f**) Al for PEO coating alone and PEO-DC coating, respectively.

In addition, the coating surface obtained by PEO followed DC exhibited the lowest pore size (typically < 1 μm in diameter) and pore density (typically < 3%) (Fig. 3b). Further, a more uniform surface is obtained after DC with thiourea than that of the PEO coating. XRD patterns were used to identify the phase structure of the coatings obtained by PEO and PEO-followed DC. XRD analyses of the PEO coatings reveal that the PEO coatings formed on AZ31 magnesium are composed of spinel (MgAl_2_O_4_) as the main phase, and MgO formed by the dehydration of Mg(OH)_2_ (Fig. 4a)^33^. Additionally, some peaks corresponding to AZ31 Mg were also detected in the XRD patterns of the untreated and treated coatings but their intensities are higher for samples with the PEO coatings. This could be attributed to the penetration of the X-ray into the AZ31 Mg samples owing to the presence of microdefects such as micropores and microcracks in the coating layer because of the inability or decreased ability of the coating to protect the AZ31 Mg (Fig. 4(a)). Diffraction peaks of the crystalline organic molecules are obviously noticed at 2θ~32° in the XRD pattern, demonstrating that the organic molecules are assembled on the inorganic surface. In order to validate the XRD results, the FT-IR spectra of the PEO and PEO-DC coatings are displayed in Fig. 4b. The ν(N-H) stretching vibration of the free inhibitor is observed at 3100–3300 cm^−1^, indicating the presence of the amine functional group on the nitrogen to inorganic surface^34^. The sharp peak at 1408 cm^−1^ is due to NH_2_ bending vibration. Moreover, a medium intensity band due to the stretching vibration (ν(C = S) = 1080 cm^−1^) of thiourea in the organic-inorganic coating is observed. The appearance of new bands in the spectra of the inhibitor-coating layer could be assigned to ν(M-O) and ν(M-N) starching vibrations as physical bonding^35^. Further, Raman spectroscopy (Fig. 4c) confirms that the fabricated organic-inorganic materials exhibit strong surface-enhanced Raman scattering activity resulting from the IL. This enhancement could be assigned to the chemical effect due to the formation of charge-transfer complexes^36^. The weak band around 466 cm^−1^ can be assigned to δ(SCN) which is slightly shifted from that of pure thiourea (401 cm^−1^), can be assigned to δ(SCN)^32^. The peak at 728 cm^−1^ corresponds to the CS stretching band, which appears at 730 cm^−1^ in the FT-IR spectrum of pure thiourea^32^. The shifts in the bending and vibration modes of sulfur bonds suggest the formation of metal with sulphur bands. The chemical composition of the organic-inorganic materials deposited on the AZ31 Mg alloy surface was further analyzed by XPS and the survey spectrum is shown in Fig. 4d. Detection of elements such as Mg, Al, O, S and N on the magnesium surface could confirm the EDS results. In addition, it could be seen that the Mg 1 s and Al 2p peaks (Fig. 4g,h) in the XPS spectrum of the PEO coating shift after the formation of organic layer on the inorganic layer by the DC method. Moreover, the core-level shifts are attributed to the changes in the eletronegativities (Pauling term) in the domain of the photoionized^37^. The higher binding energy as shown in the present work unequivocally suggest that a higher positive charge of the Mg and Al on the surface, thus indicating a higher population of Lewis acid sites on the metal surface when it has PEO-DC coating. As the electron deficiency is more on metals site so it is obvious that there will be metal-S and metal-N coordination of the samples with the PEO-DC coating.

### Electrochemical measurements

**Figure 5:**
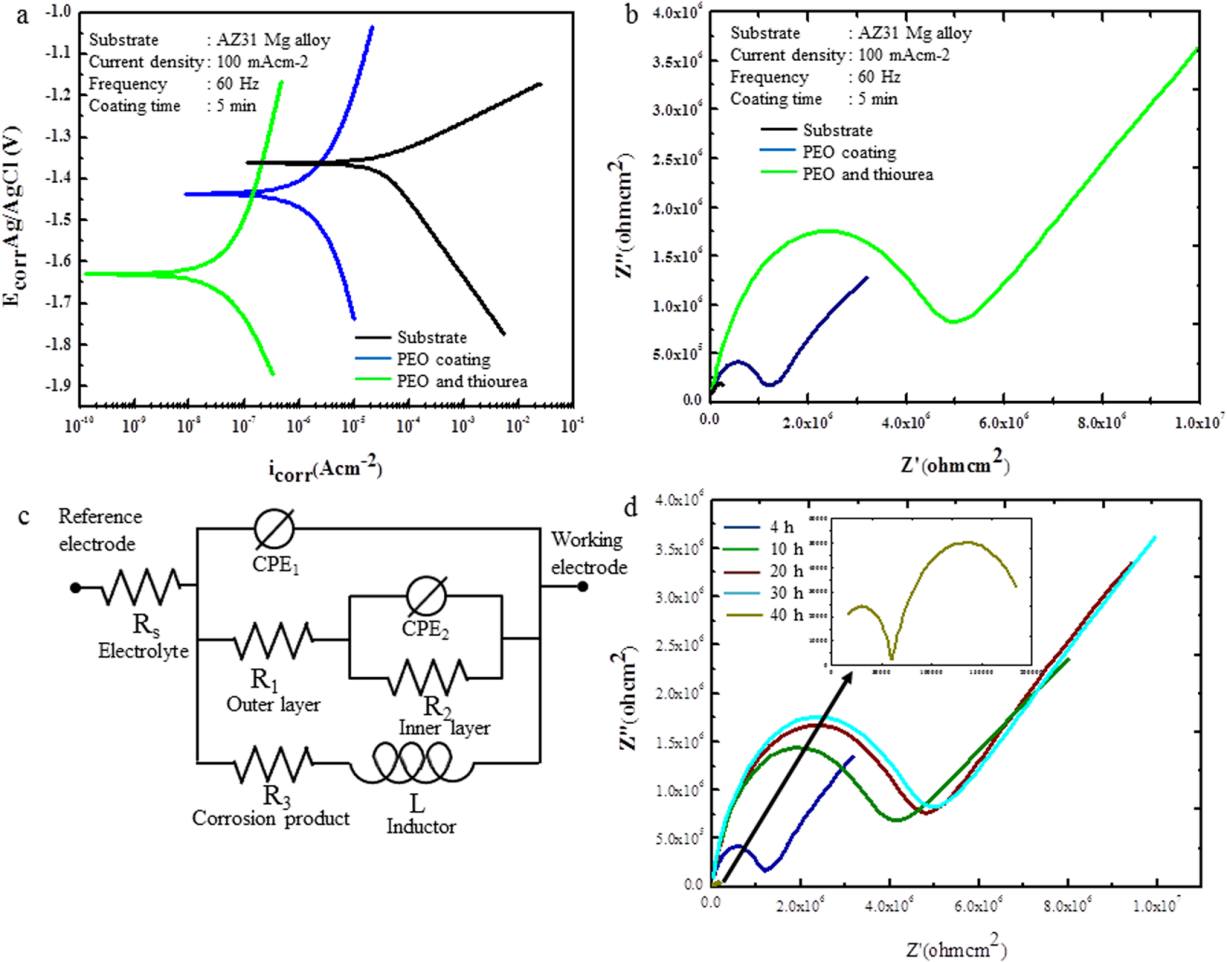
(**a**) Potentiodynamic polarization curves of the samples treated by plasma electrolytic oxidation (PEO) only and PEO followed by DC with thiourea, acquired between −0.3 to 0.4 V vs. open circuit potential in a 3.5 wt. % NaCl solution. (**b**) Electrochemical impedance spectroscopy (EIS) Nyquist plots of the samples coated with PEO only and PEO followed by DC with thiourea at 298 K. (**c**) Equivalent circuit model consisting of the organic and inorganic layers working as either resistors or condensers, and the solution resistance within the electrical cell. (**d**) EIS Nyquist plots of samples treated by PEO followed by immersion in a thiourea solution for 20 h at ambient temperature after different immersion times in a corrosive environment.

Figure 5a presents representative potentiodynamic polarization curves of the AZ31 Mg alloy with PEO coating and PEO-DC coating in a 3.5 wt% NaCl solution at 298 K. Various electrochemical parameters, including the corrosion current density (*i*_corr_), corrosion potential (*E*_corr_), anodic and cathodic Tafel slopes (*β*_a_ and *β*_c_), and inhibitors efficiency (η) were extracted from the polarization curves, and the data are listed in Table 1. Following the acquisition of the potentiodynamic polarization curves, were obtained using the Tafel extrapolation method, and the inhibition efficiency (η) is calculated by Eq. (1):

1 $$\eta  \% =\frac{{i}_{corr}^{0}-{i}_{corr}^{0}}{{i}_{corr}^{0}}\times 100$$

where *i*°_corr_ and *i*_corr_ are corrosion current densities of the unprotected and protected AZ31 Mg alloy, respectively. In the presence of the organic molecules, the *i*_corr_ values decreased sharply. Correspondingly, *η* increased in the presence thiourea, owing to a greater number of organic molecules covering the coating layer at 0.2 M. Therefore, as shown in Fig. 5a, *E*_*corr*_ values are comparable, with the variation in *E*_corr_ between the samples without and with of the organic molecules being around 85 mV, thus indicating that the organic inhibitor acts as a mixed-type inhibitor. A displacement of *E*_corr_ to less negative values in the inhibitor solution compared to the inhibitor-free PEO coating is also observed (Fig. 5a). Corrosion inhibitors that displace *E*_corr_ to less negative values are usually identified as anodic inhibitors, whereas cathodic inhibitors displace the potential to more negative values. Therefore, the obtained *E*_*corr*_ values suggest that the inhibitor has a dominant influence on the partial anodic reaction, and this phenomenon is probably caused by the barrier properties of the thiourea layer.

**Table 1:** Potentiodynamic polarization parameters of the treated samples.

Samples	*E*_corr_ (V/SCE)	*i*_corr_(μA/cm^2^)	*β*_a_(mV/dec)	*β*_c_(mV/dec)	η(%)
AZ31Mg	−1.36	32	66	−185	—
PEO coating	−1.48	0.67	498	−236	96
PEO-DC coating	−1.63	0.08	593	−363	99.7

To obtain information on the corrosion behavior of the as-fabricated composite coatings, EIS investigations were carried out for copper in a 3.5 wt% NaCl aqueous solution without and with the organic inhibitor and the results are shown in Fig. 5b. As shown in the Nyquist plots in Fig. 5b, the diameter of the curves increases remarkably with the addition of the organic layer compared with the curves of the could organic compound-free coating, indicating that this inhibitor would protect the magnesium alloy against corrosion. The results reveal improvement in the electrochemical performance of the PEO-DC coatings. This demonstrates that the PEO-DC coatings significantly improve the corrosion resistance of the magnesium alloy, which is good agreement with the results of the polarization curves. The fitted results obtained from the equivalent circuit (ECs) are described by the full lines in Fig. 5c, and the fitting parameters are listed in Table 2. The EIS spectrum of the AZ31 Mg alloy was fitted and analyzed by three well defined loops: a capacitive loop in the high frequency region, a capacitive loop in middle frequency area, and an inductive loop in the low frequency region. In this regard, the spectrum of the treated AZ31 Mg alloy consists of two loops: a capacitive one in the high frequency region and a capacitive one in the low frequency region. The capacitive loop formed at the high frequency regime is assigned to the charge transfer reaction and layer effects^38^. Moreover, the inductive loop detected in the low frequency region is related to the physical adsorption processes^39^. In the ECs, Rs is the resistance of the solution, R1 and R2 represent the resistance of the outer organic layer, and inorganic layer, respectively. R3 represents the charge transfer resistance and L denotes the corrosion behavior at low frequencies during the dissolution of the magnesium alloy. CPE1 and CPE2 represent the constant phase elements (CPEs) in parallel with R1 and R2, respectively. The data in Table 2 indicates that the addition of thiourea increased the R1 and R2 values, and the effect is improved in the presence of thiourea. Thus, results indicate that thiourea adsorption layers are formed on the inorganic surface, representing their physical adsorption on the surface of the AZ31 Mg alloy and a more effective sealing of the porous surface against mass and charge transfer^37–41^. As shown in Table 4, the resistance values of the outer part (organic layer) increased in the presence of thiourea on the inorganic surface. This increase could be assigned to the formation of an organic layer on the inorganic surface. Moreover, it is obvious that both DC coating and PEO coating show a synergistic effect leading to higher resistance of the two organic layers (organic and inorganic layers). Moreover, the CPE decreased with an increase in the thickness of the organic-inorganic coating, which could be attributed to the improvement in the surface of the electrical double layer^37^. The organic-inorganic materials between the charged AZ31 Mg surface and the NaCl solution could be considered as an electrical capacitor^41,42^. This is very important because higher resistance (Rct) indicate better inhibition performance.

**Table 2:** Electrochemical impedance parameters of the treated samples.

Sample	R_1_ (Ωcm^2^)	R_2_ (Ωcm^2^)	R_3_ (Ωcm^2^)	CPE_1_-T	CPE_1_-P	CPE_2_-T	CPE_2_-P	L (H/cm^−2^)
AZ31 Mg alloy	4 × 10^4^	3 × 10^6^	4 × 10^5^	1 × 10^−6^	0.62	3 × 10^9^	0.66	3 × 10^−4^
PEO coating	1 × 10^4^	9 × 10^8^	1 × 10^7^	3 × 10^−7^	0.40	1 × 10^−10^	0.82	136000
PEO-DC coating	4.6 × 10^6^	2.5 × 10^9^	9.2 × 10^7^	1.2 × 10^−7^	0.39	1.6 × 10^−10^	0.82	1.64 × 10^3^

In order to better recognize the organic-inorganic corrosion resistance, EIS (Fig. 5d and Table 3) was employed to assess the corrosion protection performance of the PEO in the presence of the thiourea layer. However, after only 10 h immersion, the R3 value decreased slightly to 5 × 10^7^ kΩ cm^2^, and then the R1 value reduced slightly to 3.7 × 10^6^ kΩ cm^2^ after 20 h immersion (Fig. 5d and Table 3). Upon prolonging the dipping time to 30 h, the R1, R2 and R3 values decreased further and finally reached 1.09 × 10^6^, 6.5 × 10^8^, and 1.8 × 10^7^ kΩ·cm^2^, respectively. In generally, larger diameters of the capacitive semicircle, which are denoted as R1, R2, and R3 here indicated better corrosion resistance and protection^43^. In other words, the construction of thiourea layer with PEO coating may have a negative impact on the subsequent inhibition of the Mg alloy after a long duration. After a long immersion time (30 h) (Fig. 5d), the R1, R2 and R3 values decreased remarkably, indicating that the organic-inorganic layers become thinner and the weak position damaged first, and finally, the aggressive solution reacts with the AZ31 Mg alloy^44^.

**Table 3:** EIS parameters for the Mg alloy.

Immersion time (h)	R_1_ (Ωcm^2^)	R_2_ (Ωcm^2^)	R_3_ (Ωcm^2^)	CPE_1_-T	CPE_1_-P	CPE_2_-T	CPE_2_-P	L (H/cm^−2^)
10	1.09 × 10^6^	6.5 × 10^8^	1.8 × 10^7^	2.8 × 10^−7^	0.42	1.65 × 10^−10^	0.81	2.6 × 10^5^
20	3.7 × 10^6^	2.55 × 10^9^	5.5 × 10^7^	1.4 × 10^−7^	0.33	1.47 × 10^−10^	0.83	1.01 × 10^3^
30	4.8 × 10^6^	2.5 × 10^9^	5 × 10^7^	1.2 × 10^−7^	0.42	1.95 × 10^−10^	0.80	5.03 × 10^−7^
40	6.14 × 10^4^	1.65 × 10^5^	2.5 × 10^6^	1.67 × 10^−6^	0.76	2.97 × 10^−10^	0.86	3.43

### Quantum chemical calculations

**Figure 6:**
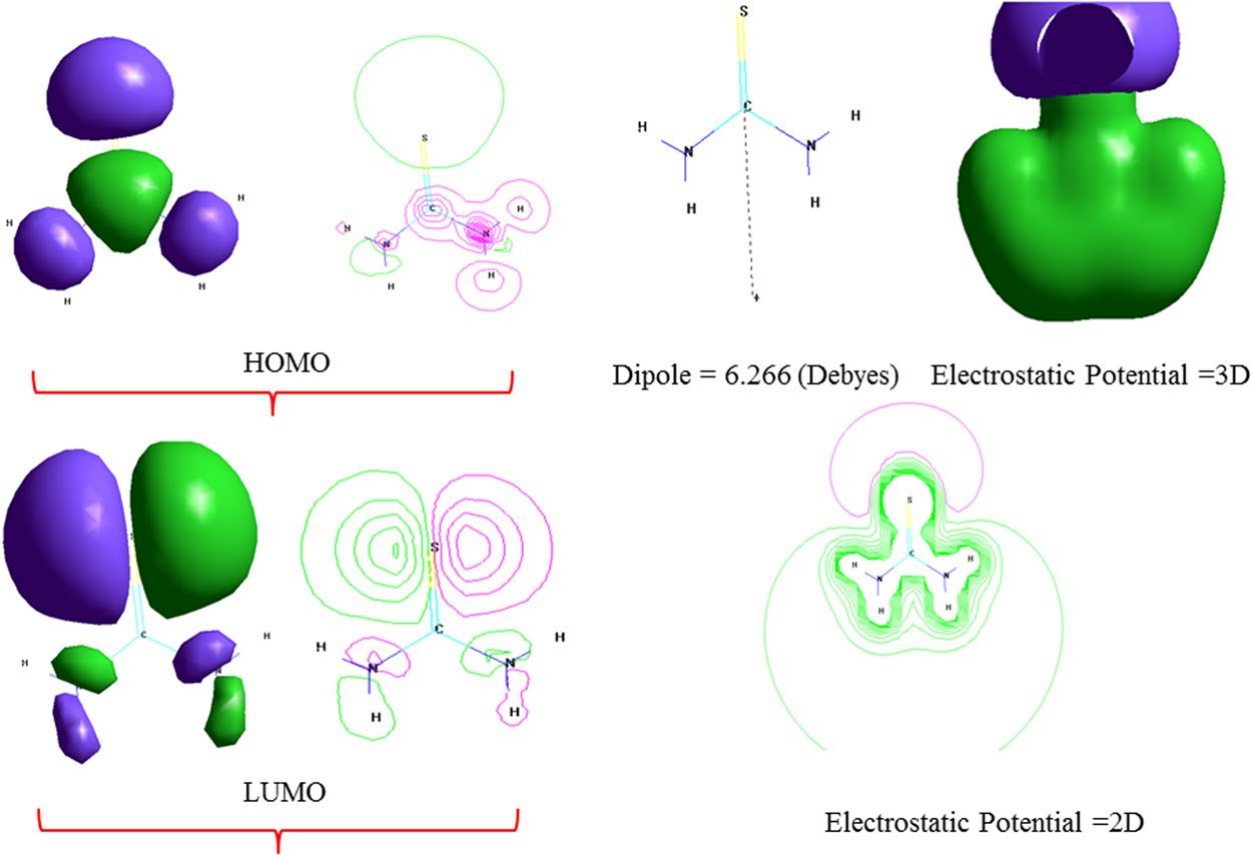
Highest occupied molecular orbitals (HOMO), lowest unoccupied molecular orbitals (LUMO) and the molecular electrostatic potential map (ESP) of thiourea.

Finally, to investigate the relationship between the structure of the inhibitor molecule and its inhibition properties, quantum chemical analyses were performed (Fig. 6). The highest occupied molecular orbital (HOMO) is often related to the electrons-donating capacity of a molecule, whereas the lowest unoccupied molecular orbital (LUMO) represents the electron-accepting ability of a molecule. The electrostatic potential (ESP) map of the studied organic inhibitor in gas phase is shown in Fig. 6^45^. Indeed, the active regions of the molecules are mainly distributed around the polar groups containing the N and S atoms. Moreover, in the ESP map, the green (negative) regions associated with the nucleophilic reactivity and the white (positive) regions related to the electrophilic reactivity are mainly distributed in the halogen atom and N atoms in the thiourea ring. Therefore, it is reasonable to suggest that thiourea contains two major adsorption sites, including two N atoms and one sulfur atom (Fig. 6). This indicates that thiourea interacts with the pores in the inorganic surface via these polar groups, and donates electrons to metal atoms with vacant d-orbitals^46^. On one hand, the higher energy of E_HOMO_ indicates that the thiourea molecules could easily donate electrons to the empty d-orbital of metal such as Mg and Al^47^. On the other hand, the lower energy of E_LUMO_ explain a higher electron accepting ability of the superficial metal^48^. Further, the ΔE value indicates the chemical stability of the metal complex which determines the interaction between the adsorbed organic compound and the inorganic surface. Smaller ΔE indicates a higher reaction activity and inhibition efficiency of the compound^49^. In the present study, the higher tendency of thiourea to be adsorbed onto the coating surface correlates with its lower ∆E value (8.3478551 eV) (Table 4). Moreover, the dipole moment (µ) is extensively used to define the polarity of an organic molecule, as a higher µ results in increased adsorption through physical forces^50,51^. Thus, µ of thiourea is 6.266 D, which is higher than that of the water molecule (1.88 D) (see Table 3). The higher µ of the inhibitor results in its adsorption on the inorganic surface, accompanied by the related physical desorption of water molecules, thereby protecting the Mg surface against the acidic solution. In addition, the high µ value indicates that thiourea is more strongly absorbed on the coating surface^49^.

**Table 4:** Quantum chemical parameters of thiourea (CS(NH_2_)_2_).

Inhibitor	E_HOMO_ (eV)	E_LOMO_ (eV)	∆E (eV)	µ(Debye)
CH_4_N_2_S	−8.618442	−0.270586	8.347855	6.266
Mg	−7.661	1.717	9.378	1.310

## Discussion

**Figure 7:**
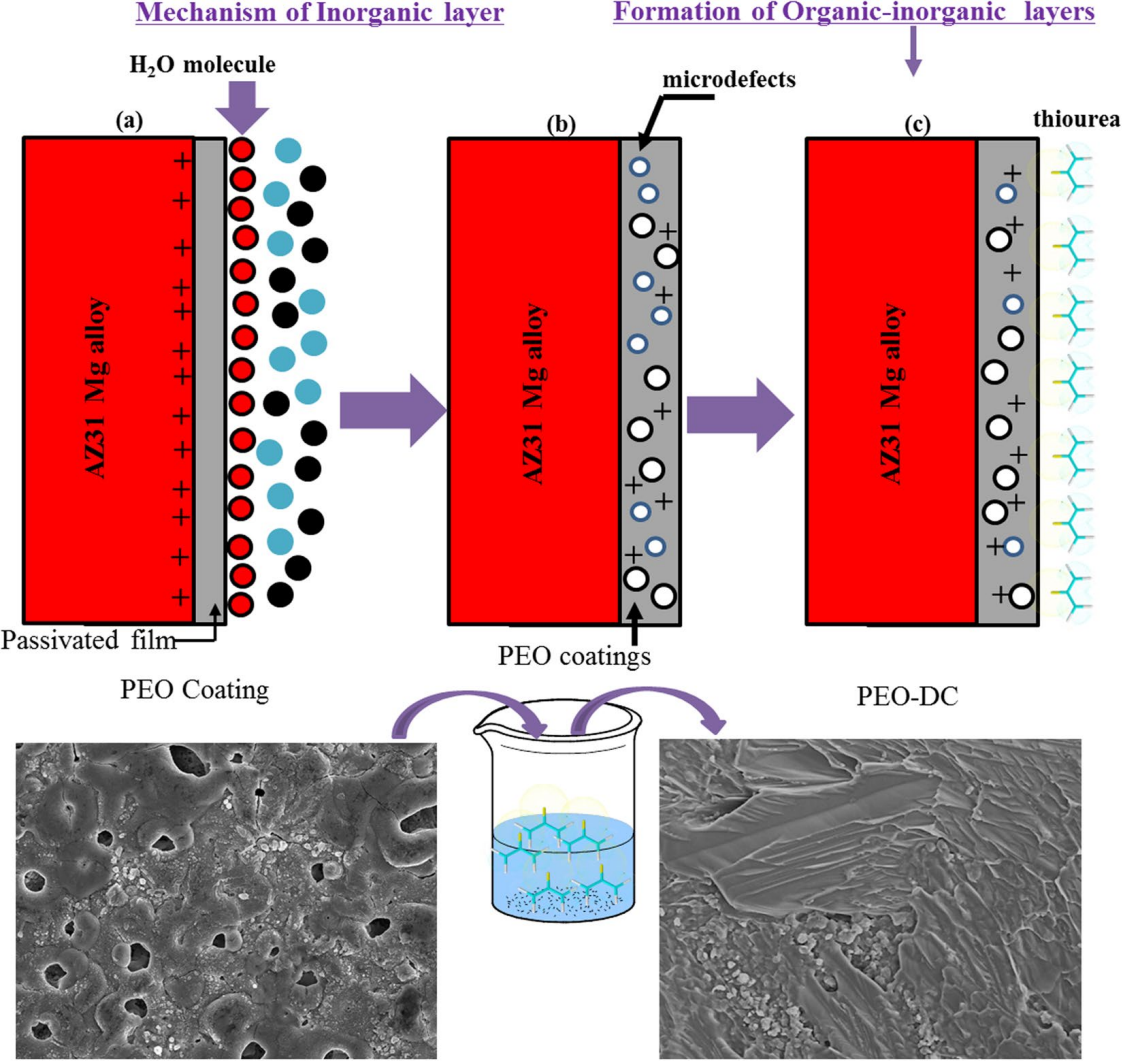
Schematic illustration of the distribution of the compounds at the anode/electrolyte interface and on the coated surface. (**a**) Plasma electrolytic oxidation (PEO) coating without hexamethylenetetramine HMT, (**b**) PEO coating in the presence of HMT, (**c**) PEO coating followed by DC in a thiourea solution.

HMT is a heterocyclic organic compound with the formula (CH_2_)_6_N_4_. It has a cage-like structure similar to adamantanes and is rather stable although dihetreo-substituted methylene groups are known to be highly reactive. Additionally, HMT was highly soluble in solvent like H_2_O, CHCl_3_, C_2_H_5_OH, and other organic solvent^52^, because HMT has three amine groups aiding for its solubility in water and it is hygroscopic in nature. Thus the effect of HMT on the PEO process gives rise to its specific physical adsorption at the interface between the AZ31 Mg anode and electrolyte in alkali solutions^53^. A model of the adsorbed layer of HMT molecules at the inorganic interface is shown in Fig. 7a. HMT possibly substitutes for the H_2_O molecular layer formed at the AZ31 Mg-solution interface in the base electrolyte (Fig. 7a), leading to a decreased solid-liquid interfacial tension. Accordingly, the unit-area adsorptive capacity of HMT molecules containing electron-donor groups close to the metal-electrolyte interface increases in number as a result of the spreadability of the electrolyte molecules at the interface. In addition, both the HMT and glycerol molecules have similar chemical structure with polar groups (-OH and -NH). That is, the HMT molecules tend to be adsorbed around the C_3_H_8_O_3_ molecules, because they have similar chemical polarity and electrostatic affinity^54^. As a result, it is reasonable to conclude that the repulsive forces diminish with the adsorption of more HMT molecules along with the glycerol molecules, leading to diminished surface tensions of these donor molecules arranged much more compactly at the metal-electrolyte interface under the electric force. These lead to the formation of dense discharge cores on the surface of the passivated inorganic coating during the PEO process, and then the passivated inorganic coating is turned into a dense inorganic coating by the activity of the electric breakdown, melting of inorganic compounds and sintering^55–57^. Further, HMT molecules adsorbed as an organic layer on the surface of the Mg alloy could increase the resistance of surface layer. This leads to the formation of active sites on the inorganic surface are divided into smaller sizes leading to decreased volume of the O_2_ bubble (Fig. 7a). In addition, for the oxide layer (Fig. 7b), the reason why it has large pores and high carbon ratio was not because it has inorganic compounds, but because the thermal decomposition of HMT could produce the following compounds; N_2_, H_2_, CH_4_, CH_3_NH_2_, H_2_O, CO_2_, HCN an oily residue, and carbon-rich residues. Therefore, the relative amount of each of the products primarily depends on the temperature of decomposition^6,58–60^.

Furthermore, Fig. 2(b) shows a SEM micrograph of coating layer-magnesium immersed for 20 h in an aqueous solution containing 3 M thiourea at room temperature. Moreover, in the presence of thiourea, the surface Fig. 1(b) seems to have a protective layer formed on the coating surface without visible cracks and pores. These results are in agreement with the electrochemical experiments (Fig. 5), wherein inhibition performance was noticed. Fig. 7 illustrates the mechanism of the formation of coating layers and adsorption/interaction between thiourea, and the coating surface/solution interface can involve the following interactions: (i) charge-transfer-type interaction between the unshared π electron pairs (S and N) and the vacant orbitals of Mg or Al surface atoms or metal oxides (chemisorption at anodic sites) (Fig. 7); (ii) electrostatic interactions between the donor atoms and the coating surface/electrolyte interface or between the protonated thiourea species (such as S atom) on the surface and adsorbed Cl^−1^_ads_ ions (physisorption at the cathodic sites); and (iii) a combination of these two (mixed type). In the studied medium, the protonation probably taking place through the S atom, as indicated in Eq. (2):

2 $${H}_{2}N-CS-N{H}_{2}\leftrightarrow {H}_{2}N-CSH-N{H}_{2}$$

According to the results of the Mulliken charge calculation, one can observe that both the neutral and protonated molecule have an excess of negative charge on the N atoms. The N atoms are probably effective adsorption centers because of they are not sterically hindered by other groups. If the metal surface/inhibitor interaction are governed by the N-atoms from thiourea, the experimental results for the inhibition efficiency of the thiourea would be higher. As for the S atom, the scenario changes after protonation while there is an excess of negative charge on the neutral thiourea derivatives, there is an excess of positive charge after protonation. However, these results must be treated with caution since it is unlikely that thiourea remains protonated it approaches the metallic surface.

## Conclusion

Finally, this study demonstrated the electrochemical coating of Mg alloy surface by the PEO method with the addition of hexamethylenetetramine followed by dip-coating with a thiourea solution.

1. The results of the microstructure analysis revealed that thiourea molecules are adsorbed to the magnesium surface and increase the carbon ratio of the inorganic layer.
2. The addition of thiourea to the inorganic layer substantially increased the corrosion protection performance of the PEO coating. This strategy not only facilitated the formation of a dense, compact coating through thiourea linkages but also ensured that the organic inhibitor rectifies the defects and micropores in the PEO coating.
3. The quantum chemical analyses provide further insight into the organic-inorganic interaction and the growth mechanism.
